# Development and validation of a regression model with nomogram for difficult video laryngoscopy in Chinese population: a prospective, single-center, and nested case-control study

**DOI:** 10.3389/fmed.2023.1197536

**Published:** 2023-09-01

**Authors:** Chenyu Jin, Bei Pei, Shuang Cao, Ningning Ji, Ming Xia, Hong Jiang

**Affiliations:** Department of Anesthesiology, Shanghai Ninth People’s Hospital, Shanghai Jiao Tong University School of Medicine, Shanghai, China

**Keywords:** difficult airway, video laryngoscopy, predictive model, Cormack-Lehane grade, Chinese population, airway rescue strategy

## Abstract

**Background:**

Airway management failure is associated with increased perioperative morbidity and mortality. Airway-related complications can be significantly reduced if difficult laryngoscopy is predicted with high accuracy. Currently, there are no large-sample studies on difficult airway assessments in Chinese populations. An airway assessment model based on the Chinese population is urgently needed to guide airway rescue strategy.

**Methods:**

This prospective nested case–control study took place in a tertiary hospital in Shanghai, China. Information on 10,549 patients was collected, and 8,375 patients were enrolled, including 7,676 patients who underwent successful laryngoscopy and 699 patients who underwent difficult laryngoscopy. The baseline characteristics, medical history, and bedside examinations were included as predictor variables. Laryngoscopy was defined as ‘successful laryngoscopy’ based on a Cormack–Lehane Grades of 1–2 and as ‘difficult laryngoscopy’ based on a Cormack–Lehane Grades of 3–4. A model was developed by incorporating risk factors and was presented in the form of a nomogram by univariate logistic regression, least absolute shrinkage and selection operator, and stepwise logistic regression. The main outcome measures were area under the curve (AUC), sensitivity, and specificity of the predictive model.

**Result:**

The AUC value of the prediction model was 0.807 (95% confidence interval [CI]: 0.787–0.828), with a sensitivity of 0.730 (95% CI, 0.690–0.769) and a specificity of 0.730 (95% CI, 0.718–0.742) in the training set. The AUC value of the prediction model was 0.829 (95% CI, 0.800–0.857), with a sensitivity of 0.784 (95% CI, 0.73–0.838) and a specificity of 0.722 (95% CI, 0.704–0.740) in the validation set.

**Conclusion:**

Our model had accurate predictive performance, good clinical utility, and good robustness for difficult laryngoscopy in the Chinese population.

## 1. Introduction

Endotracheal intubation is a crucial part of airway management for anesthesiologists (1, 2). Endotracheal intubation plays an essential role in maintaining airway safety, providing respiratory support, maintaining oxygenation, and ensuring safety. The analysis of the Anesthesia Closed Claims Project database showed that 56% of perioperative deaths or severe brain injuries were due to improper airway management, and 73% of claims were due to inappropriate airway management (3, 4). Results of the Fourth National Audit Project of the Royal College of Anesthetists and the Difficult Airway Society showed that 58% of serious airway complications were due to airway management failure (5). The 2022 American Society of Anesthesiologists Practice Guidelines for Management of the Difficult Airway recommends an airway risk assessment before every anesthesia procedure (6). The incidence of airway management failure can be significantly reduced if difficult laryngoscopy is predicted with high accuracy (7–9).

Difficult airways include difficult endotracheal intubation and difficult mask ventilation. Unanticipated difficult laryngoscopy is the main reason for difficult endotracheal intubation, which remains a significant challenge for anesthesiologists, and is associated with increased perioperative morbidity and mortality (10–13). The current widely used airway assessment tools are based on those developed for Caucasian populations (14, 15), and there are no large-sample studies of difficult airway assessment in Chinese populations. Asian populations, especially Chinese populations, differ greatly from Caucasian populations in craniomaxillofacial structure. Three-dimensional magnetic resonance imaging showed that Chinese have smaller neck circumference, smaller retropalatal airway size, and smaller tongue volume than European (16). In addition, it has been reported that Asians have higher Mallampati Score, shorter thyromental distance, larger thyromental angle, more protruding maxilla and mandible, which may contribute to higher risk of upper airway obstruction in Asians than in Caucasian populations (17, 18).

A rapid and precise airway assessment strategy is very useful in the environment of unbalanced spatial distribution of medical resources in China (19). There are two types of intubation protocols currently used by most hospitals. The first protocol is to prepare airway rescue strategy prior to all endotracheal intubations. The second protocol is to temporarily call a senior anesthesiologist and activate a follow-up airway rescue strategy after a difficult intubation has occurred. The first requires a significant drain on medical resources, while the second protocol may cause anesthesiologists to miss the “golden hour” of airway rescue. A rational airway management strategy is one that allocates limited medical resources for the most rational reasons. In other words, airway rescue strategy should accurately prepare for difficult airway, which requires an accurate airway prediction model. Hence, the current difficult airway prediction model is not suitable for the Chinese.

We conducted a prospective, nested case–control study involving 10,549 patients undergoing procedures requiring structured airway evaluation. This study was designed to identify risk factors and develop a regression model with nomogram for difficult video laryngoscopy in a Chinese population.

## 2. Methods

### 2.1. Participants

Ethical approval for this study (SH9H-2020-T176-1) was provided by the Ethics Committee of Shanghai Ninth People’s Hospital, Shanghai, China (Chairperson Prof Meng Luo) on 17 July 2020. The trial was registered prior to patient enrollment at clinicaltrials.gov (NCT04458220). Written informed consent was obtained from all subjects participating in the trial. This manuscript adheres to the applicable Strengthening the Reporting of Observational Studies in Epidemiology (STORBE) guidelines.

This prospective nested case–control study was conducted from 2 February 2021 to 28 November 2022 at the Shanghai Ninth People’s Hospital. The following inclusion criteria were used: (1) age ≥ 18 years, (2) scheduled for elective surgery, and (3) required endotracheal intubation during video laryngoscopy. The following exclusion criteria were used: (1) patients with deaf-mutism or communication disorders, (2) language deficiency or non-native language, (3) mental or central nervous system disease, (4) disturbance of consciousness, (5) severe injury (injury severity score > 15), (6) inability to follow instructions to perform standard actions, and (7) participation in other relevant clinical investigations over the past 3 months.

### 2.2. Airway assessments

The baseline characteristics and medical history of each patient were recorded before surgery. The specific airway assessment team performed preoperative bedside examinations for all patients to evaluate various airway-related parameters. The bedside examinations encompassed a comprehensive set of measurements, including: modified Mallampati test (MMT), upper lip bite test (ULBT), mandibular protrusion (MP), neck circumference (NC), cervical spine mobility (CSM), inter-incisor gap (IIG), upper incisor length (UIL), thyromental distance (TMD), sternomental distance (SMD), and hyomental distance (HMD). Additionally, we included potentially relevant and relatively important measures, such as: length of tongue (LT) (20), jaw depth (JD) (21), mandible length (ML) (22), and thyroid and hyoid distance (THD) (23). This team comprised individuals who had extensive clinical anesthesia experience, with each member having more than 3 years of experience with airway management. All members of the airway assessment team are specially trained to avoid measurement bias. They had performed over 3,000 airway assessments and tracheal intubations. The definition and classification basis of the baseline characteristics, medical history, and bedside examinations are shown in Supplementary Table S1.

### 2.3. Anesthesia protocol

All eligible patients were routinely monitored using oxygen saturation, electrocardiography, and non-invasive blood pressure measurements before induction of anesthesia. Midazolam (2–3 mg), propofol (2–3 mg/kg), fentanyl (2–4 μg/kg), and rocuronium (0.6 mg/kg) was administered before endotracheal intubation. After preoxygenation with mask-pressurized ventilation for approximately 3 min, endotracheal intubation was conducted using a video laryngoscope (video laryngoscope with Macintosh blade, such as McGrath MAC, Aircraft Medical Co., Ltd., Edinburgh, UK). Video laryngoscopy is the routine intubation device routinely used at our medical center.

Cormack–Lehane Grade assessments were performed by different well-trained researchers who were blinded to assessment results. It is essential to note that the blinding of the results was limited to the person evaluating the Cormack–Lehane Grade and did not extend to the anesthesiologist performing the intubation. Unlike direct laryngoscopy, video laryngoscopy allows all observers to clearly visualize the laryngeal exposure on the screen. During endotracheal intubation, the Cormack–Lehane Grade was evaluated (Grade 1: the glottis fully visible; Grade 2: the glottis or arytenoids partially visible; Grade 3: only the epiglottis visible; and Grade 4: epiglottis not visible) (24). In situations where the patient’s mouth opening is severely limited that the laryngoscope can barely be inserted or cannot be inserted at all, resulting in the inability to visualize the epiglottis, we classify the patient as Grade 4. Video laryngoscopy exposure with Cormack-Lehane Grades 1–2 was defined as ‘successful laryngoscopy’ and that with Cormack-Lehane Grades 3–4 was defined as ‘difficult laryngoscopy’. The Cormack–Lehane Grade was assessed by independent anesthesiologists with more than 3 years of experience with airway management. Two independent investigators jointly performed Cormack–Lehane Grade assessment during endotracheal intubation. When the assessment results of two researchers diverged, a third senior researcher made the final decision. This dual-assessor system was implemented to minimize any potential bias and to enhance the reliability of the grading process.

To avoid complications associated with difficult airway, an airway rescue strategy was applied to all participating patients throughout the research. The airway rescue strategy was as follows: (1) A senior anesthesiologist (>10 years of endotracheal intubation experience with airway management) was present throughout the induction of anesthesia; (2) Fiberoptic bronchoscopy and supraglottic airway device were available; (3) Emergency front of neck airway could be performed by oral and maxillofacial surgeon if necessary.

### 2.4. Statistical analysis

Continuous variables are reported by median (quartile) or mean ± standard deviation. Frequencies or percentages report categorical variables. The Kruskal–Wallis H test for medians, Student’s *t*-test for means, and Chi-square test for categorical variables were used for between-group comparisons. SPSS Statistics (version 25.0; IBM Inc., Armonk, NY, USA) and R Version 4.2.1 software^1^ were used for statistical analysis.

In our study, we adopted the methodology proposed by Riley et al. to determine the most suitable sample size (25). To confirm the precision of the constructed models, we performed a *post hoc* sample size calculation, taking into account a C statistic of 0.807, a prevalence of 8.35%, and predictor parameter number of 64. Using these criteria, a minimum of 5,366 instances was deemed necessary. Notably, our overall sample encompassed 8,375 patients, completely surpassing the stipulated minimum sample size requirement.

The dataset were randomly split to training and validation sets with a ratio of 7:3. The dimensionality of results were reduced by using variables with *p* ≤ 0.1 in the univariate logistic regression analysis and absolute shrinkage and selection operator (LASSO). A ten-fold cross-validation was conducted to determine the optimal parameter configuration. The non-zero coefficient features were determined based on the λ value corresponding to a standard error of the minimum distance deviation. The optimum model was established by implementing multivariable logistic regression analysis and stepwise regression. The Random Forest model is utilized to rank the importance of the variables and to demonstrate the factors which have a dominant influence in the model.

Based on the optimal model, bootstrapping validation was performed (1,000 bootstrap resamples). The constructed model was verified in the validation set by quantifying the net income within the threshold probability range. A calibration curve was developed to evaluate the model calibration. Receiver operating characteristic (ROC) curve analysis was conducted to evaluate differential efficacy, and a decision curve analysis (DCA) was plotted to evaluate the clinical application value of the model. Delong test was performed to compare differences in predictive performance between the two models. In the decision curve analysis, “intervention for all” indicates that airway rescue strategy was applied to all patients, and “intervention for none” indicates that the temporary airway rescue strategy was applied only after the emergence of a difficult airway.

## 3. Results

**Figure 1:**
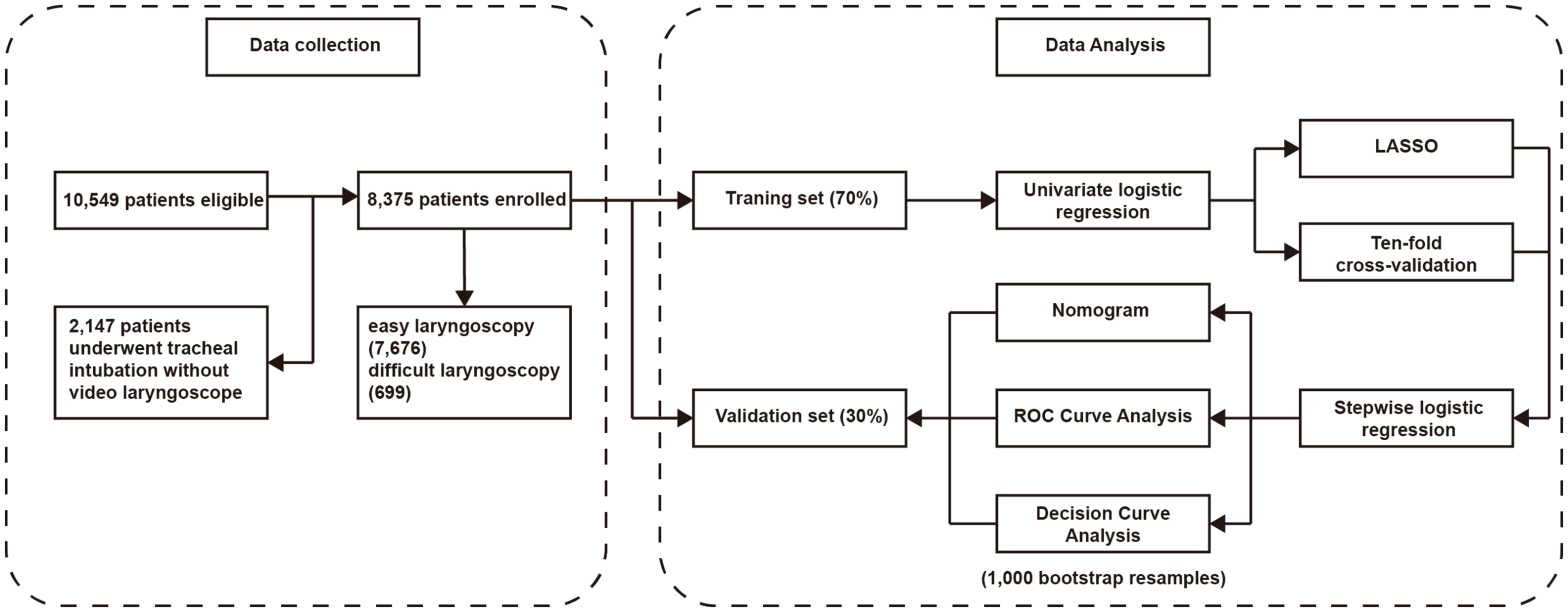
The flow chart of this research. LASSO, least absolute shrinkage and selection operator; ROC, receiver operating characteristic.

A total of 10,549 patients underwent detailed consultation and bedside examination, and their information was recorded before endotracheal intubation. A total of 2,147 patients were excluded because they underwent endotracheal intubation without video laryngoscopy (such as using direct laryngoscope, fiberoptic bronchoscope) at first attempt. Finally, 8,375 patients were enrolled, including 7,676 patients with successful laryngoscopy, and 699 patients with difficult laryngoscopy. In comparison to patients with successful laryngoscopy, those experiencing difficult laryngoscopy exhibit several significant differences. These include higher age, BMI, incidence of alcohol consumption, incidence of smoking, American Society of Anesthesiologists Physical Status (ASA-PS) level, incidence of cardiovascular disease history, diabetes history, rhinitis, snoring, difficult intubation history, radiotherapy history, surgery history, maxillofacial tumor/trauma, buck teeth, and head and neck scar. Additionally, patients with difficult laryngoscopy tend to have higher levels of MMT, ULBT, MP, CSM, and NC, while exhibiting lower values in LT, IIG, THD, HMD, TMD, SMD, ML, and JD. Notably, there were 232 (33.2%) difficult intubations in patients with difficult laryngoscopy compared to 104 (1.4%) in patients with successful laryngoscopy. The results of baseline characteristics of included patients are shown in Table 1. A flow chart of the study is shown in Figure 1. The characteristics of the patients in the training and validation sets are shown in Supplementary Tables S2, S3, respectively.

**Table 1:** The baseline characteristics of included patients.

	Overall	Successful laryngoscopy	Difficult laryngoscopy	*p*
Number	8,374	7,675	699	
Difficult intubation, *n* (%)	336 (4.0)	104 (1.4)	232 (33.2)	<0.001
Age, [mean (SD)]	38.9 (15.5)	38.1 (15.3)	48.1 (15.4)	<0.001
Gender (male), *n* (%)	3,856 (46.0)	3,485 (45.4)	371 (53.1)	<0.001
BMI, [mean (SD)]	22.4 (3.6)	22.4 (3.5)	22.9 (3.9)	<0.001
Chinese nationality (non-Han), *n* (%)	175 (2.1)	164 (2.1)	11 (1.6)	0.391
Alcohol consumption, *n* (%)	1,839 (22.0)	1,646 (21.4)	193 (27.6)	<0.001
Smoking, *n* (%)	1,778 (21.2)	1,575 (20.5)	203 (29.0)	<0.001
Beard, *n* (%)	7 (0.1)	6 (0.1)	1 (0.1)	1
ASA-PS 1:2:3, *n* (%)	5,671:2,588:115 (67.7:30.9:1.4)	5,362:2,216:97 (69.9:28.9:1.3)	309:372:18 (44.2:53.2:2.6)	<0.001
Snoring, *n* (%)	3,768 (45.0)	3,361 (43.8)	407 (58.2)	<0.001
DI history, *n* (%)	15 (0.2)	9 (0.1)	6 (0.9)	<0.001
Radiotherapy, *n* (%)	243 (2.9)	155 (2.0)	88 (12.6)	<0.001
Surgery history, *n* (%)	4,166 (49.7)	3,757 (49.0)	409 (58.5)	<0.001
History of mandible operation, *n* (%)	309 (3.7)	285 (3.7)	24 (3.4)	0.786
Maxillofacial tumor, *n* (%)	412 (4.9)	314 (4.1)	98 (14.0)	<0.001
Maxillofacial trauma, *n* (%)	120 (1.4)	100 (1.3)	20 (2.9)	0.002
Buck teeth, *n* (%)	255 (3.0)	217 (2.8)	38 (5.4)	<0.001
Head and neck scar, *n* (%)	130 (1.6)	100 (1.3)	30 (4.3)	<0.001
MMT 1:2:3:4, *n* (%)	2,512:1,919:3,373:570 (30.0:22.9:40.3:6.8)	2,429:1,790:3,091:365 (31.6:23.3:40.3:4.8)	83:129:282:205 (11.9:18.5:40.3:29.3)	<0.001
ULBT 1:2:3, *n* (%)	5,923:1,689:762 (70.7:20.2:9.1)	5,602:1,506:567 (73.0:19.6:7.4)	321:183:195 (45.9:26.2:27.9)	<0.001
MP 1:2:3, *n* (%)	7,201:946:227 (86.0:11.3:2.7)	6,742:779:154 (87.8:10.1:2.0)	459:167:73 (65.7:23.9:10.4)	<0.001
CSM 1:2:3, *n* (%)	8,077:236:61 (96.4:2.8:0.7)	7,467:172:36 (97.3:2.2:0.5)	610:64:25 (87.0:9.2:3.6)	<0.001
NC [mean (SD)]	35.6 (4.0)	35.6 (4.0)	36.5 (4.2)	<0.001
LT [mean (SD)]	4.4 (1.01)	4.4 (0.94)	3.7 (1.42)	<0.001
IIG [mean (SD)]	4.0 (0.9)	4.1 (0.8)	3.1 (1.3)	<0.001
UIL [mean (SD)]	0.9 (0.4)	0.9 (0.4)	0.9 (0.4)	0.857
THD [mean (SD)]	4.3 (1.1)	4.3 (1.1)	3.9 (1.1)	<0.001
HMD [mean (SD)]	4.4 (1.0)	4.4 (1.0)	4.1 (1.0)	<0.001
TMD [mean (SD)]	9.2 (1.4)	9.3 (1.4)	8.6 (1.4)	<0.001
SMD [mean (SD)]	16.6 (2.3)	16.7 (2.3)	15.5 (2.2)	<0.001
ML [mean (SD)]	9.8 (1.3)	9.8 (1.2)	9.6 (1.4)	0.004
JD [mean (SD)]	3.7 (0.6)	3.7 (0.6)	3.5 (0.6)	<0.001

SD, standard deviation; BMI, body mass index; ASA-PS, American Society of Anesthesiologists Physical Status; MMT, modified Mallampati test; ULBT, upper lip bite test; MP, mandibular protrusion; NC, neck circumference; LT, length of tongue; JD, jaw depth; ML, mandible length; CSM, cervical spine mobility; IIG, inter-incisor gap; UIL, upper incisor length; TMD, thyromental distance; SMD, sternomental distance; THD, thyroid and hyoid distance; HMD, hyomental distance.

Firstly, a univariate logistic regression analysis was performed for the initial screening of the variables. We defined *p* < 0.1 as the cut-off value, and 30 variables related to difficult laryngoscopy were determined for further analysis, as shown in Table 2.

**Table 2:** Results of the univariate logistic regression analysis.

Variables	OR (95% CI)	*p*
Age (year)	1.04 (1.03, 1.05)	<0.001
Gender (Male)	1.43 (1.18, 1.72)	<0.001
Education (level 1 as reference)		
2	0.64 (0.51, 0.79)	<0.001
3	0.31 (0.24, 0.39)	<0.001
BMI (kg/m^2^)	1.05 (1.02, 1.07)	<0.001
Alcohol consumption (Yes)	1.55 (1.25, 1.90)	<0.001
Smoking (Yes)	1.69 (1.37, 2.07)	<0.001
ASA-PS (Non-ASA I)	2.74 (2.27, 3.32)	<0.001
History of cardiovascular diseases (Yes)	2.33 (1.85, 2.91)	<0.001
History of diabetes (Yes)	2.12 (1.40, 3.10)	<0.001
History of snoring (Yes)	1.83 (1.51, 2.22)	<0.001
History of radiotherapy (Yes)	6.49 (4.59, 9.08)	<0.001
History of surgery (Yes)	1.46 (1.21, 1.76)	<0.001
History of rhinitis (Yes)	0.79 (0.64, 0.97)	0.026
Head and neck scar (Yes)	3.53 (2.07, 5.76)	<0.001
History of maxillofacial tumors (Yes)	3.32 (2.44, 4.45)	<0.001
History of maxillofacial trauma (Yes)	2.33 (1.25, 4.04)	0.004
Buck teeth (Yes)	2.10 (1.36, 3.13)	<0.001
Tongue hypertrophy (Yes)	3.09 (0.70, 9.94)	0.084
MMT (level 1 as reference)		
2	2.50 (1.78, 3.54)	<0.001
3	2.96 (2.19, 4.07)	<0.001
4	18.34 (13.04, 26.14)	<0.001
ULBT (level 1 as reference)		
2	2.26 (1.79, 2.83)	<0.001
3	6.00 (4.71, 7.62)	<0.001
MP (level 1 as reference)		
2	3.24 (2.57, 4.07)	<0.001
3	6.70 (4.63, 9.57)	<0.001
NC (cm)	1.06 (1.04, 1.09)	<0.001
LT (cm)	0.54 (0.49, 0.59)	<0.001
JD (cm)	0.79 (0.66, 0.94)	0.008
CSM (level > 90° as reference)		
80–90°	5.73 (3.91, 8.27)	<0.001
<80°	9.11 (4.86, 16.75)	<0.001
IIG (cm)	0.39 (0.36, 0.43)	<0.001
TMD (cm)	0.75 (0.70, 0.80)	<0.001
SMD (cm)	0.80 (0.77, 0.84)	<0.001
THD (cm)	0.72 (0.65, 0.79)	<0.001
HMD (cm)	0.72 (0.65, 0.79)	<0.001

BMI, body mass index; ASA-PS, American Society of Anesthesiologists Physical Status; MMT, modified Mallampati test; ULBT, upper lip bite test; MP, mandibular protrusion; NC, neck circumference; LT, length of tongue; JD, jaw depth; ML, mandible length; CSM, cervical spine mobility; IIG, inter-incisor gap; UIL, upper incisor length; TMD, thyromental distance; SMD, sternomental distance; THD, thyroid and hyoid distance; HMD, hyomental distance; Education 1, Junior high school education or below; Education 2, High school education or above, without a bachelor’s degree; Education 3, Bachelor, Master, or Doctor degree.

**Figure 2:**
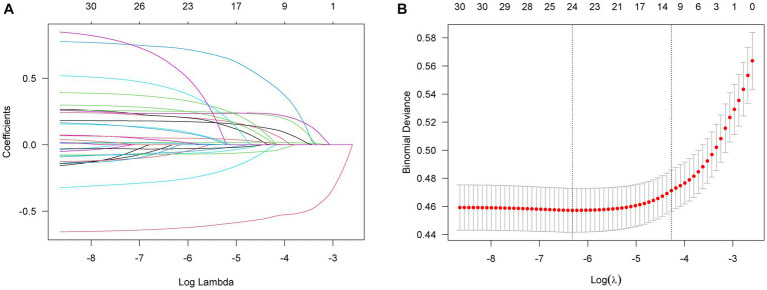
The univariate logistic regression analysis by LASSO. **(A)** 30 variables with *p* ≤ 0.1. **(B)** Variables selection in LASSO regression. LASSO, Least Absolute Shrinkage and Selection Operator.

**Figure 3:**
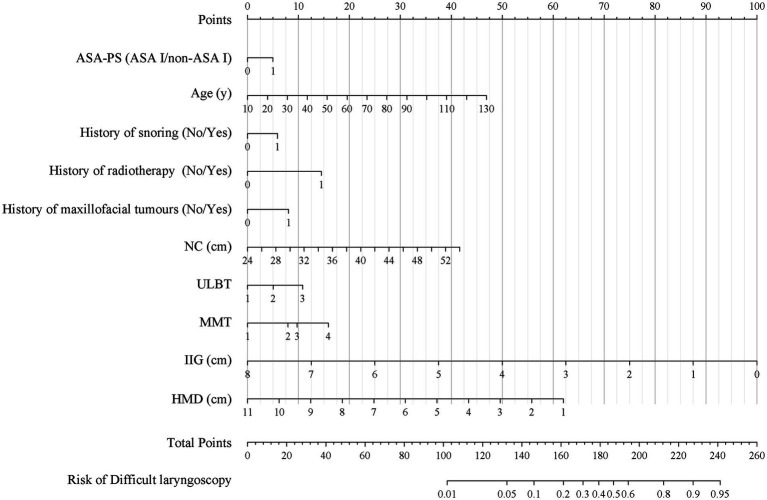
The nomogram for difficult laryngoscopy; The nomogram for difficult laryngoscopy was developed with ASA-PS (American Society of Anesthesiologists Physical Status), age, history of snoring, history of radiotherapy, history of maxillofacial tumors; NC, neck circumference; ULBT, upper lip bite test; MMT, modified Mallampati test; IIG, inter-incisor gap; and HMD, hyomental distance; ASA-PS 0, ASA I classification; ASA-PS 1, non-ASA I classification; History of snoring 0, No; History of snoring 1, Yes; History of radiotherapy 0, No; History of radiotherapy 1, Yes; History of maxillofacial tumors 0, No; History of maxillofacial tumors 1, Yes.

**Figure 4:**
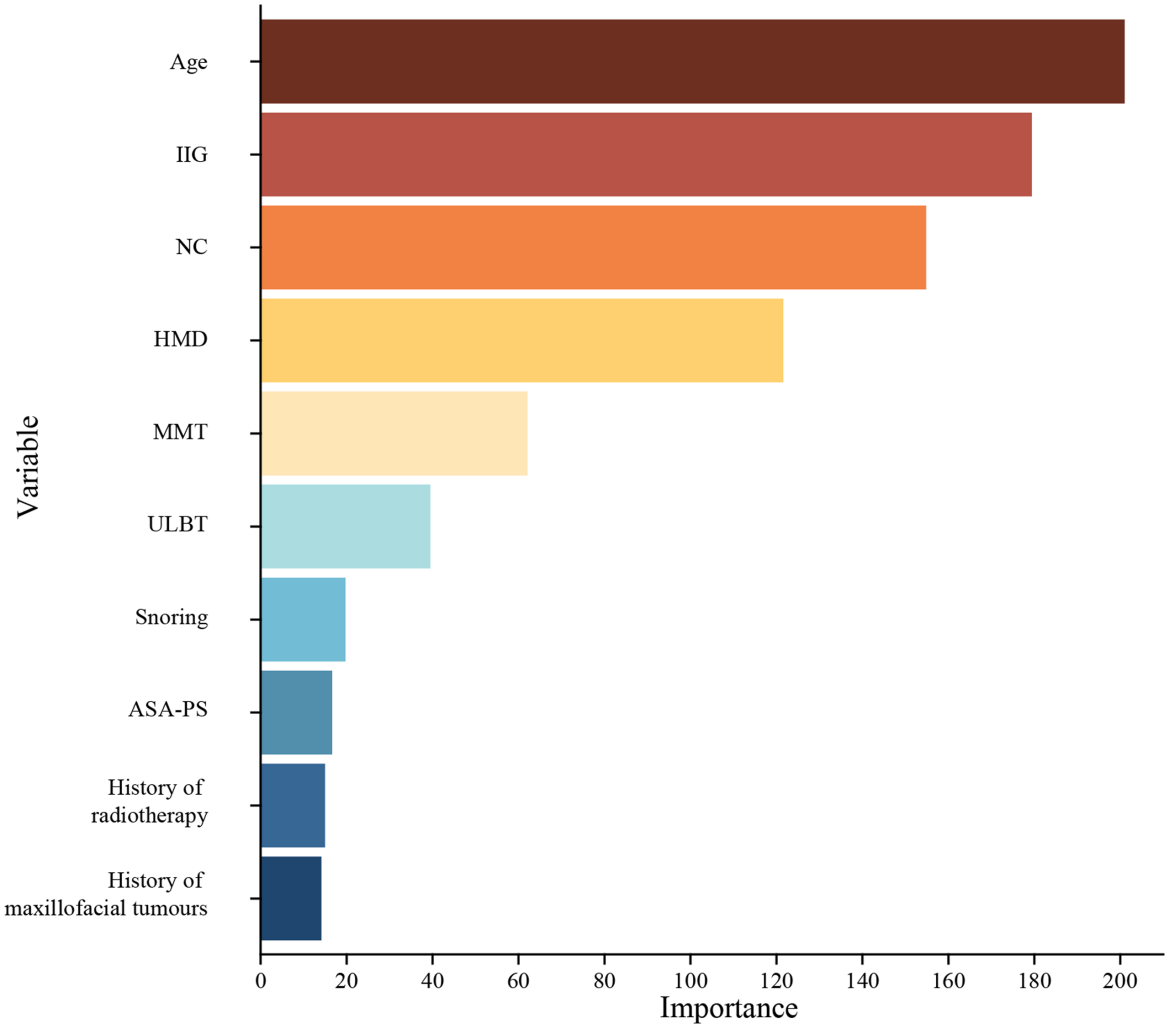
The variable importance ranking. IIG, inter-incisor gap; NC, neck circumference; HMD, hyomental distance; MMT, modified Mallampati test; ULBT, upper lip bite test; ASA-PS, American Society of Anesthesiologists Physical Status.

Next, we conducted further screening on the initially identified variables using LASSO analysis and stepwise regression. After LASSO analysis and stepwise regression, a ten-variable analysis was conducted for the optimum prediction model (Figure 2). These features included ASA-PS, age, history of snoring, history of radiotherapy of head and neck region, history of maxillofacial tumors, NC, ULBT, MMT, IIG, and HMD (Table 3). The optimum model was developed by integrating risk factors and was illustrated as a nomogram (Figure 3). In addition, the Random Forest model was used to calculate the importance of the included factors, and the results showed that the five most important factors were: age, IIG, NC, HMD, and MMT. The results of variable importance ranking are shown in Figure 4.

**Table 3:** Prediction factors of difficult laryngoscopy.

Variable	OR (95% CI)	*p*
ASA-PS (Non-ASA I)	1.31 (1.03, 1.66)	0.025
Age (year)	1.02 (1.01, 1.03)	<0.001
History of snoring (Yes)	1.37 (1.10, 1.71)	0.005
History of radiotherapy (Yes)	2.19 (1.44, 3.30)	<0.001
History of maxillofacial tumors (Yes)	1.55 (1.07, 2.20)	0.018
NC (cm)	1.08 (1.05, 1.11)	<0.001
ULBT (level 1 as reference)		
2	1.31 (1.02, 1.69)	0.035
3	1.80 (1.33, 2.41)	<0.001
MMT (level 1 as reference)		
2	1.69 (1.19, 2.43)	0.004
3	1.54 (1.11, 2.15)	0.010
4	2.36 (1.51, 3.70)	<0.001
IIG (cm)	0.51 (0.45, 0.58)	<0.001
HMD (cm)	0.72 (0.64, 0.80)	<0.001

ASA-PS, American Society of Anesthesiologists Physical Status; NC, neck circumference; ULBT, upper lip bite test; MMT, modified Mallampati test; IIG, inter-incisor gap; HMD, hyomental distance.

**Figure 5:**
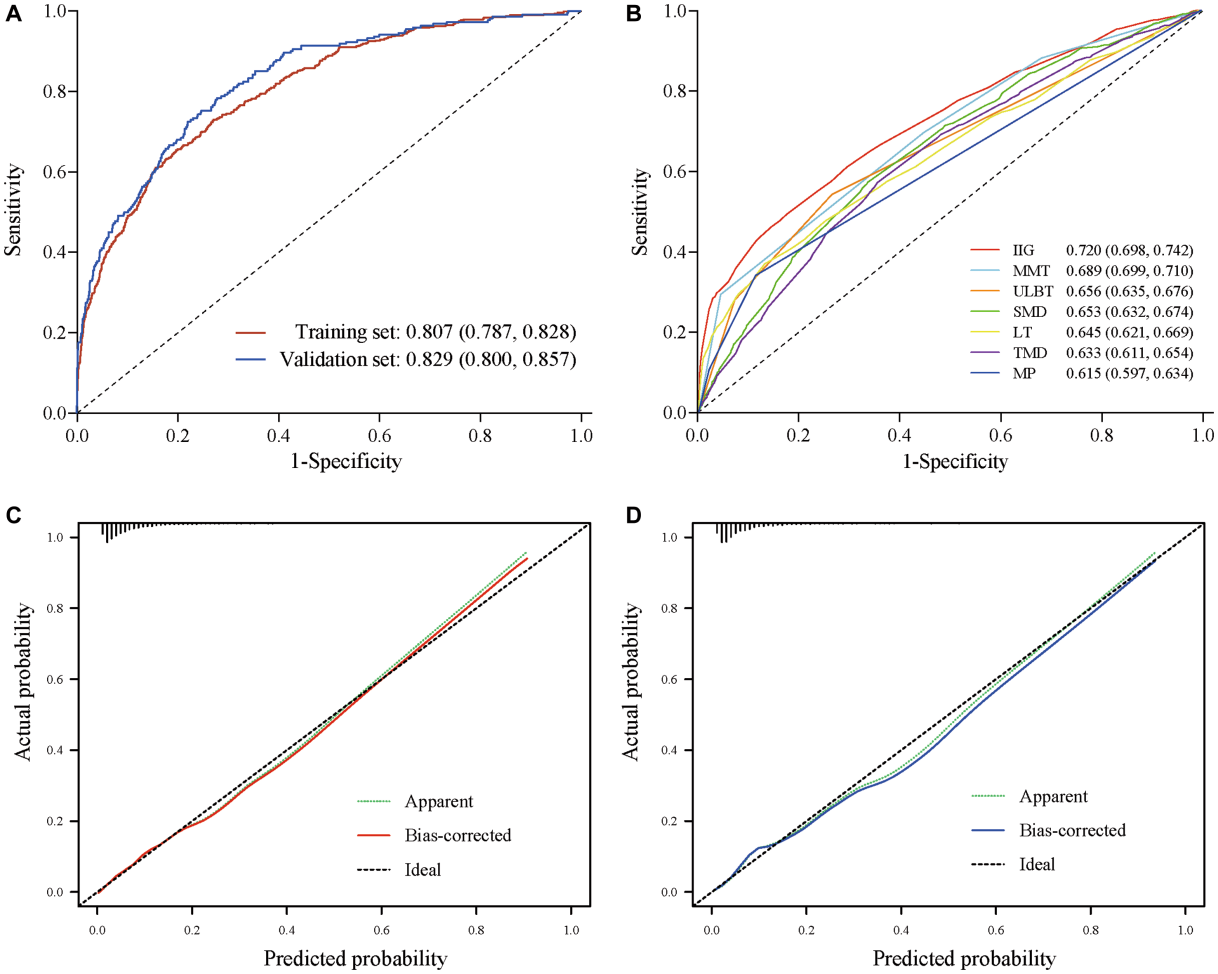
ROC and calibration curve. **(A)** ROC curve of regression model in training and validation set. **(B)** ROC curve of bedside examinations with AUC > 0.6. **(C)** Calibration curves in training set. **(D)** Calibration curves nomogram in validation set.

ROC curve analysis was conducted to evaluate the differential efficacy of the model for difficult laryngoscopies. The AUC (area under the curve) value of the prediction model was 0.807 [95% confidence interval (CI): 0.787–0.828] with a sensitivity of 0.730 (95% CI, 0.690–0.769) and a specificity of 0.730 (95% CI, 0.718–0.742) in the training set. The AUC value of the prediction model was 0.829 (95% CI, 0.800–0.857), with a sensitivity of 0.784 (95% CI, 0.730–0.838) and a specificity of 0.722 (95% CI, 0.704–0.740) in the validation set (Figure 5A).

ROC curve analysis was also conducted to evaluate the differential efficacy of bedside examinations for difficult laryngoscopies. The results showed that IIG, MMT, ULBT, SMD, LT, TMD, and MP had AUC values above 0.6 (Figure 5B). The best predictor among bedside examinations is IIG, which has an AUC of 0.720 (95% CI, 0.698, 0.742), a sensitivity of 0.510 (95% CI, 0.419, 0.675) and a specificity of 0.824 (95% CI, 0.650, 0.890). The comprehensive scores showed an AUC of 0.668 (95% CI, 0.646, 0.689) for Wilson Score and an AUC of 0.709 (95% CI, 0.690, 0.729) for El Ganzouri risk index (EGRI). In addition, the prediction performance of our prediction model was significantly superior to the widely used airway assessment tools, including individual bedside examination and comprehensive score (*p* < 0.001). The predictive performance of all bedside examinations is shown in Table 4.

**Table 4:** Predictive performance of bedside examinations.

Bedside examinations	Cut off value	AUC (95% CI)	Sensitivity (95% CI)	Specificity (95% CI)	Accuracy (95% CI)
IIG (cm)*	3.45	0.720 (0.698, 0.742)	0.510 (0.419, 0.675)	0.824 (0.650, 0.890)	0.797 (0.652, 0.852)
MMT*	3.5	0.689 (0.669, 0.710)	0.326 (0.270, 0.730)	0.953 (0.544, 0.961)	0.897 (0.558, 0.906)
ULBT*	1.5	0.656 (0.635, 0.676)	0.541 (0.504, 0.578)	0.735 (0.725, 0.745)	0.719 (0.709, 0.728)
SMD (cm)*	15.95	0.653 (0.632, 0.674)	0.582 (0.506, 0.729)	0.663 (0.507, 0.729)	0.656 (0.525, 0.711)
LT (cm)*	3.45	0.645 (0.621, 0.669)	0.374 (0.332, 0.502)	0.868 (0.745, 0.885)	0.828 (0.725, 0.842)
TMD (cm)*	8.95	0.633 (0.611, 0.654)	0.586 (0.494, 0.720)	0.642 (0.509, 0.732)	0.636 (0.524, 0.710)
MP*	1.5	0.615 (0.597, 0.634)	0.339 (0.305, 0.374)	0.887 (0.880, 0.895)	0.842 (0.835, 0.849)
THD (cm)*	4.15	0.590 (0.567, 0.612)	0.663 (0.334, 0.732)	0.490 (0.429, 0.80)	0.503 (0.451, 0.763)
HMD (cm)*	4.35	0.589 (0.566, 0.611)	0.651 (0.327, 0.696)	0.490 (0.467, 0.799)	0.503 (0.483, 0.762)
JD (cm)*	3.35	0.567 (0.544, 0.591)	0.425 (0.275, 0.667)	0.707 (0.471, 0.828)	0.683 (0.485, 0.784)
NC (cm)*	34.25	0.561 (0.539, 0.583)	0.686 (0.605, 0.746)	0.445 (0.390, 0.520)	0.465 (0.418, 0.528)
CSM*	1.5	0.551 (0.539, 0.583)	0.126 (0.102, 0.151)	0.976 (0.973, 0.979)	0.906 (0.902, 0.909)
ML (cm)*	9.15	0.538 (0.514, 0.562)	0.382 (0.175, 0.489)	0.701 (0.616, 0.894)	0.675 (0.603, 0.835)
UIL (cm)*	1.05	0.488 (0.465, 0.511)	0.140 (0.044, 0.962)	0.896 (0.056, 0.978)	0.834 (0.132, 0.901)
Wilson Score*	1.5	0.668 (0.646, 0.689)	0.854 (0.575, 0.862)	0.418 (0.383, 0.697)	0.817 (0.584, 0.825)
EGRI*	2.5	0.709 (0.690, 0.729)	0.743 (0.461, 0.753)	0.568 (0.534, 0.831)	0.728 (0.491, 0.738)

AUC, area under curve; CI, confidence interval; MMT, modified Mallampati test; ULBT, upper lip bite test; MP, mandibular protrusion; NC, neck circumference; LT, length of tongue; JD, jaw depth; ML, mandible length; CSM, cervical spine mobility; IIG, inter-incisor gap; UIL, upper incisor length; TMD, thyromental distance; SMD, sternomental distance; THD, thyroid and hyoid distance; HMD, hyomental distance; EGRI, El Ganzouri risk index. ^*^Compared to the optimum model, *p* < 0.001.

We compared several large-sample studies due to the lack of airway-related data specifically available for the Caucasian population (26–28). A comprehensive analysis of various airway-related parameters between the Chinese and Caucasian populations was conducted. The Chinese population displayed notably higher MMT scores, a lower incidence of patients with an increased neck circumference, reduced limited TMD/MP, a lower obesity rate, and a lower incidence in the presence of beard compared to the Caucasian population. The results of difference between the Caucasian and Chinese population in airway-related parameters are shown in Supplementary Table S4.

**Figure 6:**
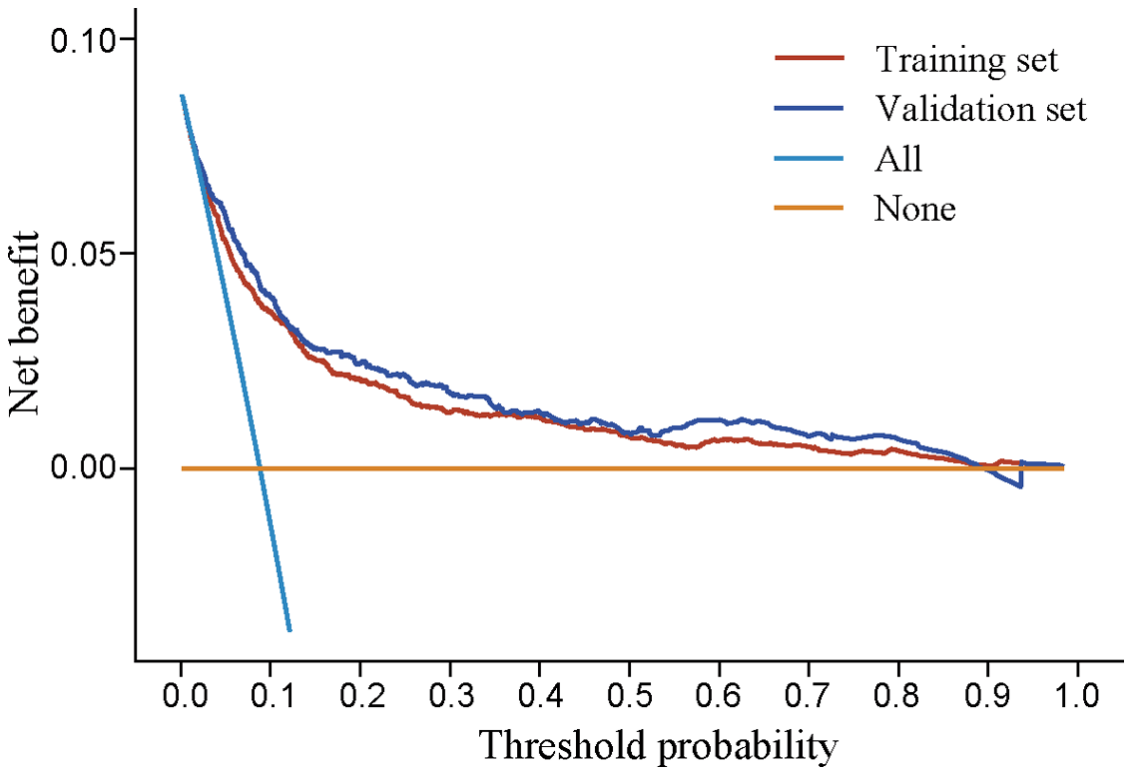
Decision curve assessment in training and validation set. All: airway rescue strategy preparation was applied to all patients; None: the temporary airway rescue strategy was applied only after the emergence of difficult airway.

The calibration plot revealed good predictive accuracy between the actual and predicted probabilities in the training and validation set (Figures 5C,D). The DCA showed intervening (airway rescue strategy preparation) on patients according to the prediction model leads to higher benefit than the alternative strategies of airway rescue preparation for all patients, or temporarily airway rescue strategy when difficult airway occurred (Figure 6).

## 4. Discussion

Unanticipated difficult laryngoscopy is the main reason for undetected difficult airways, and is a great challenge for anesthesiologists. The results showed that patients with high level ASA-PS, advanced age, history of snoring, radiotherapy of head and neck region, maxillofacial tumors, increased NC (>34.3 cm), ULBT (>1 level), MMT (> 3 level), limited IIG (<3.5 cm), and limited HMD (<4.4 cm) helps to predict difficult laryngoscopy. Furthermore, the inclusion of these 10 variables in the prediction model for difficult laryngoscopy showed an AUC value of 0.807 in training model and an AUC value of 0.829 in validation model, which is significantly superior to the widely used airway assessment tools. The Random Forest model was used to calculate the importance of the included factors, and the results showed that the five most important factors were: age, IIG, NC, HMD, and MMT. These five factors have dominant influences in our model. Therefore, this comprehensive model holds promise in aiding anesthesiologists in identifying and managing challenging airway situations more effectively.

The current widely used airway assessment tools are based on those developed for Caucasian populations. During our research, we meticulously examined airway-related parameters in both Chinese and Caucasian populations, drawing comparisons with data from several large-sample studies (26–28). The findings highlighted noteworthy distinctions between these two groups, providing valuable insights into potential anatomical variations and their implications for airway management and clinical approaches. Notably, the Chinese population displayed higher MMT scores, a lower incidence of patients with an increased neck circumference, reduced limited TMD/MP, a lower obesity rate, and a lower incidence in the presence of beard compared to the Caucasian population. Such a difference is consistent with the results of previous studies (16–18), and these differences may be related to the fact that currently widely used airway assessment methods are not applicable to the Chinese population.

To our knowledge, this study is the first to identify risk factors and develop a regression model with nomogram for difficult laryngoscopy based on a large sample of the Chinese population. Unanticipated difficult laryngoscopy is the main reason for difficult endotracheal intubation (10), which is consistent with our findings (33.2% in difficult laryngoscopy group vs. 1.4% in successful laryngoscopy group). The model developed in our study incorporated 10 predictive factors, including 5 medical histories and 5 bedside examinations. It is convenient and efficient to collect information of medical history. Generally, the medical history information will be recorded in electronic medical record system, and anesthesiologists need only minor confirmation. Furthermore, it takes only less than 2 min to perform the 5 bedside examinations. Based on our regression model, the anesthesiologists can perform airway evaluation accurately by collecting the most valuable information in the limited time. When a patient is evaluated preoperatively for a possible “difficult laryngoscopy,” an airway rescue strategy should be activated. A senior anesthesiologist should replace the junior anesthesiologist for endotracheal intubation, and airway rescue equipment such as fiberoptic bronchoscope or supraglottic airway device should be available.

The results of the study showed that the predictive performance of the regression model was superior to that of a single bedside examination. This result was anticipated because the regression model already included multiple bedside examinations with high AUC value. A single bedside examination reflects only a single or few characteristics of the patient. For example, IIG represents only the difficulty of placing the laryngoscope. However, the regression model covers 5 medical histories and 5 bedside examinations and enables a comprehensive representation of multiple airway characteristics of the patient.

Video laryngoscopy is more widely used than direct laryngoscopy in China. This study focuses on the Cormack–Lehane Grade during video laryngoscopy, which is in accordance with the medical situation in China. Firstly, video laryngoscopy is associated with improved pharynx exposure compared with direct laryngoscopy (29). Secondly, the ability to view the laryngeal structures on a monitor screen during video laryngoscopy enables better communication, facilitating shared decision-making. In contrast, direct laryngoscopy restricts the view to the operator alone, limiting the opportunity for immediate feedback and shared visualization. However, the difficult airway caused by difficult laryngoscopy should not be underestimated during the use of video laryngoscopy. Using predictive models developed based on direct laryngoscopy can lead to an excessive false positive rate, and therefore increase the burden of anesthesia efforts. In our study, we specifically focused on evaluating the Cormack-Lehane Grade during video laryngoscopy. This choice aligns with the medical practices prevalent in China. The Cormack-Lehane grading system is commonly used and has been traditionally based on direct laryngoscopy. Nevertheless, it is also widely accepted for its application during video laryngoscopy, and it remains the best classification criterion for our study due to its representativeness and clinical value. It is important to recognize that Cormack-Lehane grade I and II indicates successful laryngoscopy, whereas grade III and IV indicates failed laryngoscopy (30).

The results suggested that the inclusion of ASA-PS and age in the prediction model helps to predict difficult laryngoscopy. These two variables suggest that the patient’s health level or comorbidity might correlate with difficult laryngoscopy. Patients of ASA I classification have a difficult laryngoscopy rate of 5.5% (311/5,672), and for patients of the non-ASA I classification, this is 14.4% (388/2,314). Many other studies have also shown that ASA-PS and age are risk factors for difficult laryngoscopy/airway (31, 32), which is consistent with our results.

The results suggested that the inclusion of history of snoring in the prediction model helps to predict difficult laryngoscopy. A history of snoring, an important clinical symptom of obstructive sleep apnea (OSA), was associated with difficult laryngoscopy in our patient cohort. Snoring is a common predictor of difficult laryngoscopy (33, 34), and it is often found in other airway assessment tools for difficult airways, such as the STOP-Bang questionnaire (35). The 2022 ASA Practice Guidelines for Management of the Difficult Airway consider both snoring and OSA to be important risk factors for a difficult airway (6). However, in clinical practice, many patients are unaware of whether they are suffering from OSA because the gold standard for OSA diagnosis is polysomnography.

The results suggested that the inclusion of history of maxillofacial tumors and radiotherapy of head and neck region in the prediction model helps to predict difficult laryngoscopy, which is also consistent with previous studies (36–38). Maxillofacial tumors are often associated with intraoral tumor occupancy, restricted mouth opening, pathological jaw fractures, and upper airway obstruction. Difficult airways occur significantly more frequently in oral and maxillofacial surgery than in other surgical procedures. The incidence of difficult laryngoscopy varied from 8.9 to 15.4% in previous studies (38–40). A history of radiotherapy leads to structural changes in the airway, such as oedema, fibrosis, or even necrosis, and these radiation-induced airway changes may affect the tracheal cartilage, jawbone, and soft tissue structures (36, 37).

NC is the circumference measured at the level of the thyroid cartilage, and the results showed that it was also helpful in predicting difficult laryngoscopy. Several studies have shown that obesity and NC are independent predictors of difficult airways (41). A correlation analysis showed an increased risk of difficult intubation when the neck circumference was >42 cm (41). In addition, there is a seven-fold increase in the risk of difficult intubation when the NC increases from 40 to 60 cm (42).

ULBT, MMT, and IIG are common predictive measures for difficult airway management and have been included in the El-Ganzouri Risk Index (15). The ULBT assesses the mobility of the mandible by whether the patient can bite the upper lip with the lower incisors. MMT is the most frequently used clinical test. Studies have shown that modified Mallampati scores of 3–4 have good accuracy for predicting difficult laryngoscopy (43). A short IIG represents impaired mouth opening. It was impossible to insert a laryngoscope blade when the patient’s mouth opening was severely impaired. Lifting the laryngoscope also became problematic when the IIG was sufficient to place the laryngoscope blade.

A shorter HMD was also helpful in predicting difficult laryngoscopies. It has been found that the position of the hyoid bone could be an essential anatomical factor contributing to a difficult airway (44). Other studies have similarly concluded that HMD is an effective predictor, and the cut-off value of HMD ranges from 3.5 cm to 5.5 cm (44–46). It is notable that HMD was included in the model instead of TMD, which may be due to several reasons. First, HMD may capture additional relevant characteristics, such as the volume of the pharyngeal cavity. Second, TMD could potentially be influenced by various external factors. TMD’s interpretability may be influenced by external factors, such as the heightened thyroid cartilage levels observed in males.

There are some limitations to the current research. First, this was a single-center study with inherent limitations, such as a limited patient population, which would lead to an unavoidable risk of bias and limit the robustness of our results in other populations. Second, this study was conducted at a general hospital renowned for its specialized departments, particularly in oral and maxillofacial surgery and ENT (Ear, Nose, and Throat) surgery, which resulted in a higher incidence of difficult laryngoscopy (8.35%, 699/8375). Previous study with large samples also showed that patients undergoing surgery in the departments of maxillofacial (8.9%) and ENT (7.4%) have the highest rates of difficult laryngoscopy, while the overall difficult laryngoscopy in the study was 4.9% (38), and such results are consistent with the results in our center. Third, the Cormack–Lehane Grade only reflects the exposure level of the pharynx during intubation, not difficult intubation, or difficult airway. Nevertheless, difficult laryngoscopy is commonly used in clinical practice and Cormack–Lehane Grade is the most commonly definition, which represents the risk of a difficult airway or difficult intubation (47). Besides the limitations, our study has several advantages. This study is the first to develop a regression model for difficult laryngoscopy based on a large sample of the Chinese population. Our results showed that the regression models had high AUC values, sensitivity, and specificity in both the training and validation groups. The calibration plot revealed good predictive accuracy between the actual and predicted probabilities, which represented good robustness and value for general application. In the future, we plan to conduct multicenter studies to improve the generalizability of the Chinese population.

In conclusion, our regression model with nomogram had accurate predictive performance, good clinical utility, and good robustness for difficult laryngoscopy in the Chinese population. Airway rescue strategy preparation according to the prediction model leads to high benefit.

## Data availability statement

The raw data supporting the conclusions of this article will be made available by the authors, without undue reservation.

## Ethics statement

The studies involving humans were approved by the Ethics Committee of Shanghai Ninth People’s Hospital, Shanghai, China. The studies were conducted in accordance with the local legislation and institutional requirements. The participants provided their written informed consent to participate in this study.

## Author contributions

MX and HJ contributed to the conception of the study. CJ, BP, SC, and MX contributed to the methodology of the study. CJ and BP contributed to the collection and assembly of data. CJ and NJ contributed to the data analysis and interpretation. CJ, NJ, MX, and HJ contributed to the writing, review, and editing of the manuscript. All authors contributed to manuscript revision, read, and approved the submitted version.

## Funding

This study was supported by the Clinical Research Program of 9th People’s Hospital, Shanghai Jiao Tong University School of Medicine (No. JYJC202133).

## Conflict of interest

The authors declare that the research was conducted in the absence of any commercial or financial relationships that could be construed as a potential conflict of interest.

## Publisher’s note

All claims expressed in this article are solely those of the authors and do not necessarily represent those of their affiliated organizations, or those of the publisher, the editors and the reviewers. Any product that may be evaluated in this article, or claim that may be made by its manufacturer, is not guaranteed or endorsed by the publisher.

## Figures and Tables

**Table:** 

STORBE	Strengthening the Reporting of Observational Studies in Epidemiology
MMT	Modified Mallampati test
ULBT	Upper lip bite test; MP, mandibular protrusion
NC	Neck circumference
LT	Length of tongue
JD	Jaw depth
ML	Mandible length
CSM	Cervical spine mobility
IIG	Inter-incisor gap
UIL	Upper incisor length
TMD	Thyromental distance
SMD	Sternomental distance
THD	Thyroid and hyoid distance
HMD	Hyomental distance
LASSO	Least absolute shrinkage and selection operator
ROC	Receiver operating characteristic
DCA	Decision curve analysis
ASA-PS	American Society of Anesthesiologists Physical Status
CI	Confidence interval
AUC	Area under the curve
OSA	Obstructive sleep apnea

## Footnotes

^1^ https://www.r-project.org/
