# Supplementary material for: Development and validation of a regression model with nomogram for difficult video laryngoscopy in Chinese population: a prospective, single-center, and nested case-control study
Source: Front Med (Lausanne). 2023 Sep 1;10:1197536. doi: 10.3389/fmed.2023.1197536 (PMC10505806; doi:10.3389/fmed.2023.1197536)
Supplement: Supplementary file 1 [file Table_1.DOCX]

**Supplemental Table S1: Description of Airway Assessments**

| **Variable** | **detail** |
| --- | --- |
| **Baseline characteristics** |  |
| Surgical department | the surgical department of the patient, such as Department of Oral & Maxillofacial Surgery |
| Chinese nationality | the Chinese nationality of the patient, such as the Han nationality |
| Age | Age of the patient |
| Gender | Gender of patient |
| Education | Education level of the patient  Education 1: Junior high school education or below;  Education 2: High school education or above, without a bachelor's degree;  Education 3: Bachelor, Master, or Doctor degree |
| BMI | BMI of the patient |
| Alcohol consumption | Whether the patient had a drinking habit. |
| Smoking | Whether the patient had a smoking habit. |
| Beard | Whether the patient has a long beard. |
| ASA-PS | ASA-Physical Status:  ASA-PS 1: in good health;  ASA-PS 2: with mild comorbidities;  ASA-PS 3: with severe comorbidities and limited activity ability;  ASA-PS 4: with severe comorbidities and no activity ability |
| **Medical history** |  |
| History of cardiovascular diseases | Whether the patient has a previous history of cardiovascular diseases. |
| History of diabetes | Whether the patient has a previous history of diabetes. |
| History of cranial diseases | Whether the patient has a previous history of cranial diseases. |
| History of respiratory diseases | Whether the patient has a previous history of respiratory diseases. |
| History of thyroid-related diseases | Whether the patient has a previous history of thyroid-related diseases. |
| History of liver-related diseases | Whether the patient has a previous history of liver-related diseases. |
| History of gastrointestinal diseases | Whether the patient has a previous history of gastrointestinal diseases. |
| History of spine-related diseases | Whether the patient has a previous history of spine-related diseases. |
| History of urological diseases | Whether the patient has a previous history of urological diseases. |
| History of rheumatic and immunological diseases | Whether the patient has a previous history of rheumatic and immunological diseases. |
| History of gynecological and breast diseases | Whether the patient has a previous history of gynecological and breast diseases. |
| History of allergy | Whether the patient has a previous history of allergy. |
| History of ENT diseases | Whether the patient has a previous history of ENT diseases. |
| History of mental illness | Whether the patient has a previous history of mental illness. |
| History of hematologic diseases | Whether the patient has a previous history of hematologic diseases. |
| History of snoring | Whether the patient has a previous history of snoring. |
| History of difficult intubation | Whether the patient has a previous history of difficult intubation. |
| History of radiotherapy | Whether the patient has a previous history of radiotherapy. |
| History of surgery | Whether the patient has a previous history of surgery history. |
| History of mandible operation | Whether the patient has a previous history of mandible operation history. |
| History of rhinitis | Whether the patient has a previous history of rhinitis. |
| Nasal congestion | Whether the patient is currently suffering from nasal congestion. |
| Head and neck scar | Whether the patient is currently suffering from head and neck scar. |
| History of maxillofacial tumours | Whether the patient has a previous history of maxillofacial tumours. |
| History of maxillofacial trauma | Whether the patient has a previous history of maxillofacial trauma. |
| buck teeth | Whether the patient currently has buck teeth. |
| tongue hypertrophy | Whether the patient is currently suffering from tongue hypertrophy. |
| Laryngeal edema | Whether the patient is currently suffering from laryngeal edema. |
| epiglottis swelling | Whether the patient is currently suffering from epiglottis swelling. |
| Tonsillar hypertrophy | Whether the patient is currently suffering from tonsillar hypertrophy. |
| laryngospasm | Whether the patient is currently suffering from laryngospasm. |
| bronchospasm | Whether the patient is currently suffering from bronchospasm. |
| airway obstruction | Whether the patient is currently suffering from airway obstruction. |
| pneumothorax | Whether the patient is currently suffering from pneumothorax. |
| Thoracic deformity | Whether the patient is currently suffering from thoracic deformity. |
| other related syndromes | Whether the patient is currently suffering from other syndromes (including Pierre-Robin Syndrome, Klippel-Feil Syndrome, Treacher-Collins Syndrome, Down’s syndrome, etc.). |
| **bedside examinations** |  |
| MMT | modified Mallampati test: a grading system used to rate the visibility of the structures in the oropharynx, including the uvula, faucial pillars, and soft palate when the mouth is opened;  MMT 1: the soft palate, the pharyngopalatine arch, the uvula, and the hard palate are visible;  MMT 2: the soft palate, the uvula, and the hard palate are visible;  MMT 3: the soft palate and the hard palate are visible;  MMT 4: only hard palate is visible; |
| ULBT | upper lip bite test: measured by asking patients to bite their upper lip with their lower incisors;  UBLT 1: the lower incisors extend beyond the vermilion border of the upper lip;  UBLT 2: the lower incisors bite the lip but cannot extend above the vermilion border;  UBLT 3: the lower incisors cannot bite the upper lip; |
| MP | mandibular protrusion: the range of movement of the mandible;  MP 1: The lower incisors extend beyond the upper incisors;  MP 2: The lower incisors are flush with the upper incisors;  MP 3: The lower incisors extend within the upper incisors; |
| NC | neck circumference: circumference of the neck at the level of the thyroid cartilage. |
| LT | length of tongue: the maximum length of the tongue outside the mouth. |
| JD | jaw depth: the distance of the edge of lower lip and chin |
| ML | mandible length: the length of mandible. |
| CSM | cervical spine mobility  CSM 1: > 90°  CSM 2: 80-90°  CSM 3: < 80° |
| IIG | inter-incisor gap: the maximal distance between the upper and lower incisors. |
| UIL | upper incisor length: the length of upper incisor. |
| TMD | thyromental distance: the distance between the upper-most border of the thyroid cartilage and the mentum with the neck extended. |
| SMD | sternomental distance: the distance between the upper border of the sternum and the tip of the jaw with the neck fully extended |
| THD | thyroid and hyoid distance: the distance between the thyroid and the hyoid with the neck extended |
| HMD | hyomental distance: the distance between the hyoid bone and the mentum with the neck extended |

BMI: body mass index; ASA-PS: American Society of Anesthesiologists Physical Status; MMT: modified Mallampati test; ULBT: upper lip bite test; MP: mandibular protrusion; NC; neck circumference; LT: length of tongue; JD: jaw depth; ML: mandible length; CSM: cervical spine mobility; IIG: inter-incisor gap; UIL: upper incisor length; TMD: thyromental distance; SMD: sternomental distance; THD: thyroid and hyoid distance; HMD: hyomental distance.
