# Supplementary material for: Development and validation of a regression model with nomogram for difficult video laryngoscopy in Chinese population: a prospective, single-center, and nested case-control study
Source: Front Med (Lausanne). 2023 Sep 1;10:1197536. doi: 10.3389/fmed.2023.1197536 (PMC10505806; doi:10.3389/fmed.2023.1197536)
Supplement: Supplementary file 2 [file Table_2.DOCX]

**Supplemental Table S2: Description of the training set**

|  | **Training set** | | | | |  |
| --- | --- | --- | --- | --- | --- | --- |
|  | **Easy laryngoscopy** | **Difficult laryngoscopy** | | **p** | |  |
|  | ***N=5384*** | ***N=477*** | |  | |  |
| **Baseline characteristics** |  | |  | |  | |
| Surgical department: |  |  | | . | |  |
| ENT | 895 (16.62%) | 57 (11.95%) | |  | |  |
| Oral & Maxillofacial Surgery | 3668 (68.13%) | 371 (77.78%) | |  | |  |
| Ambulatory ward | 332 (6.17%) | 17 (3.56%) | |  | |  |
| Neurosurgery | 79 (1.47%) | 3 (0.63%) | |  | |  |
| Ophthalmology | 76 (1.41%) | 1 (0.21%) | |  | |  |
| Plastic and Reconstructive Surgery | 312 (5.79%) | 27 (5.66%) | |  | |  |
| Others | 22 (0.41%) | 1 (0.21%) | |  | |  |
| Chinese nationality: |  |  | | 0.886 | |  |
| The Han nationality | 5271 (97.90%) | 468 (98.11%) | |  | |  |
| Others | 113 (2.10%) | 9 (1.89%) | |  | |  |
| Age | 37.95±15.18 | 48.27±15.35 | | <0.001 | |  |
| Gender: |  |  | | <0.001 | |  |
| Female | 2936 (54.53%) | 218 (45.70%) | |  | |  |
| Male | 2448 (45.47%) | 259 (54.30%) | |  | |  |
| Education: |  |  | | <0.001 | |  |
| 1 | 1280 (23.77%) | 197 (41.30%) | |  | |  |
| 2 | 1693 (31.45%) | 166 (34.80%) | |  | |  |
| 3 | 2411 (44.78%) | 114 (23.90%) | |  | |  |
| BMI | 22.38±3.51 | 22.99±3.85 | | 0.001 | |  |
| Alcohol consumption: |  |  | | <0.001 | |  |
| No | 4216 (78.31%) | 334 (70.02%) | |  | |  |
| Yes | 1168 (21.69%) | 143 (29.98%) | |  | |  |
| Smoking: |  |  | | <0.001 | |  |
| No | 4277 (79.44%) | 332 (69.60%) | |  | |  |
| Yes | 1107 (20.56%) | 145 (30.40%) | |  | |  |
| Beard: |  |  | | 0.448 | |  |
| No | 5378 (99.89%) | 476 (99.79%) | |  | |  |
| Yes | 6 (0.11%) | 1 (0.21%) | |  | |  |
| ASA-PS: |  |  | | <0.001 | |  |
| 1 | 3747 (69.60%) | 217 (45.49%) | |  | |  |
| 2 | 1637 (30.40%) | 260 (54.51%) | |  | |  |
| **Medical history** |  | |  | |  | |
| History of cardiovascular diseases: |  |  | | <0.001 | |  |
| No | 4750 (88.22%) | 364 (76.31%) | |  | |  |
| Yes | 634 (11.78%) | 113 (23.69%) | |  | |  |
| History of diabetes: |  |  | | <0.001 | |  |
| No | 5213 (96.82%) | 446 (93.50%) | |  | |  |
| Yes | 171 (3.18%) | 31 (6.50%) | |  | |  |
| History of cranial diseases: |  |  | | 0.176 | |  |
| No | 5369 (99.72%) | 474 (99.37%) | |  | |  |
| Yes | 15 (0.28%) | 3 (0.63%) | |  | |  |
| History of respiratory diseases: |  |  | | 0.384 | |  |
| No | 5293 (98.31%) | 472 (98.95%) | |  | |  |
| Yes | 91 (1.69%) | 5 (1.05%) | |  | |  |
| History of thyroid-related diseases: |  |  | | 0.262 | |  |
| No | 5283 (98.12%) | 472 (98.95%) | |  | |  |
| Yes | 101 (1.88%) | 5 (1.05%) | |  | |  |
| History of liver-related diseases: |  |  | | 1.000 | |  |
| No | 5318 (98.77%) | 471 (98.74%) | |  | |  |
| Yes | 66 (1.23%) | 6 (1.26%) | |  | |  |
| History of gastrointestinal diseases: |  |  | | 0.944 | |  |
| No | 5308 (98.59%) | 471 (98.74%) | |  | |  |
| Yes | 76 (1.41%) | 6 (1.26%) | |  | |  |
| History of spine-related diseases: |  |  | | 0.324 | |  |
| No | 5329 (98.98%) | 475 (99.58%) | |  | |  |
| Yes | 55 (1.02%) | 2 (0.42%) | |  | |  |
| History of urological diseases: |  |  | | 0.197 | |  |
| No | 5368 (99.70%) | 474 (99.37%) | |  | |  |
| Yes | 16 (0.30%) | 3 (0.63%) | |  | |  |
| History of rheumatic and immunological diseases: |  |  | | 1.000 | |  |
| No | 5352 (99.41%) | 475 (99.58%) | |  | |  |
| Yes | 32 (0.59%) | 2 (0.42%) | |  | |  |
| History of gynecological and breast diseases: |  |  | | 0.288 | |  |
| No | 5381 (99.94%) | 476 (99.79%) | |  | |  |
| Yes | 3 (0.06%) | 1 (0.21%) | |  | |  |
| History of allergy: |  |  | | 0.616 | |  |
| No | 5372 (99.78%) | 477 (100.00%) | |  | |  |
| Yes | 12 (0.22%) | 0 (0.00%) | |  | |  |
| History of ENT diseases: |  |  | | 0.721 | |  |
| No | 5358 (99.52%) | 476 (99.79%) | |  | |  |
| Yes | 26 (0.48%) | 1 (0.21%) | |  | |  |
| History of mental illness: |  |  | | 1.000 | |  |
| No | 5365 (99.65%) | 476 (99.79%) | |  | |  |
| Yes | 19 (0.35%) | 1 (0.21%) | |  | |  |
| History of hematologic diseases: |  |  | | 0.675 | |  |
| No | 5366 (99.67%) | 475 (99.58%) | |  | |  |
| Yes | 18 (0.33%) | 2 (0.42%) | |  | |  |
| History of snoring: |  |  | | <0.001 | |  |
| No | 2984 (55.42%) | 193 (40.46%) | |  | |  |
| Yes | 2400 (44.58%) | 284 (59.54%) | |  | |  |
| History of difficult intubation: |  |  | | 0.225 | |  |
| No | 5382 (99.96%) | 476 (99.79%) | |  | |  |
| Yes | 2 (0.04%) | 1 (0.21%) | |  | |  |
| History of radiotherapy: |  |  | | <0.001 | |  |
| No | 5278 (98.03%) | 422 (88.47%) | |  | |  |
| Yes | 106 (1.97%) | 55 (11.53%) | |  | |  |
| History of surgery: |  |  | | <0.001 | |  |
| No | 2793 (51.88%) | 203 (42.56%) | |  | |  |
| Yes | 2591 (48.12%) | 274 (57.44%) | |  | |  |
| History of mandible operation: |  |  | | 0.544 | |  |
| No | 5191 (96.42%) | 463 (97.06%) | |  | |  |
| Yes | 193 (3.58%) | 14 (2.94%) | |  | |  |
| History of rhinitis: |  |  | | 0.029 | |  |
| No | 3590 (66.68%) | 342 (71.70%) | |  | |  |
| Yes | 1794 (33.32%) | 135 (28.30%) | |  | |  |
| Nasal congestion: |  |  | | 0.566 | |  |
| No | 4909 (91.18%) | 431 (90.36%) | |  | |  |
| Left | 126 (2.34%) | 10 (2.10%) | |  | |  |
| Right | 276 (5.13%) | 31 (6.50%) | |  | |  |
| Bilateral | 73 (1.36%) | 5 (1.05%) | |  | |  |
| Head and neck scar: |  |  | | <0.001 | |  |
| No | 5318 (98.77%) | 457 (95.81%) | |  | |  |
| Yes | 66 (1.23%) | 20 (4.19%) | |  | |  |
| History of maxillofacial tumours: |  |  | | <0.001 | |  |
| No | 5156 (95.77%) | 416 (87.21%) | |  | |  |
| Yes | 228 (4.23%) | 61 (12.79%) | |  | |  |
| History of maxillofacial trauma: |  |  | | 0.006 | |  |
| No | 5315 (98.72%) | 463 (97.06%) | |  | |  |
| Yes | 69 (1.28%) | 14 (2.94%) | |  | |  |
| Buck teeth: |  |  | | 0.001 | |  |
| No | 5229 (97.12%) | 449 (94.13%) | |  | |  |
| Yes | 155 (2.88%) | 28 (5.87%) | |  | |  |
| Tongue hypertrophy: |  |  | | 0.100 | |  |
| No | 5373 (99.80%) | 474 (99.37%) | |  | |  |
| Yes | 11 (0.20%) | 3 (0.63%) | |  | |  |
| Laryngeal edema |  |  | | 1.000 | |  |
| No | 5384 (100%) | 477 (100%) | |  | |  |
| Yes | 0 (0%) | 0 (0%) | |  | |  |
| Epiglottis swelling: |  |  | | 0.197 | |  |
| No | 5368 (99.70%) | 474 (99.37%) | |  | |  |
| Yes | 16 (0.30%) | 3 (0.63%) | |  | |  |
| Tonsillar hypertrophy: |  |  | | 0.133 | |  |
| No | 5378 (99.89%) | 475 (99.58%) | |  | |  |
| Yes | 6 (0.11%) | 2 (0.42%) | |  | |  |
| laryngospasm |  | |  | | 1.000 | |
| No | 5384 (100%) | | 477 (100%) | |  | |
| Yes | 0 (0%) | | 0 (0%) | |  | |
| bronchospasm |  | |  | | 1.000 | |
| No | 5384 (100%) | | 477 (100%) | |  | |
| Yes | 0 (0%) | | 0 (0%) | |  | |
| airway obstruction |  | |  | | 1.000 | |
| No | 5384 (100%) | | 477 (100%) | |  | |
| Yes | 0 (0%) | | 0 (0%) | |  | |
| pneumothorax: |  |  | | 1.000 | |  |
| No | 5381 (99.94%) | 477 (100.00%) | |  | |  |
| Yes | 3 (0.06%) | 0 (0.00%) | |  | |  |
| Thoracic deformity |  |  | | 1.000 | |  |
| No | 2291 (100%) | 222 (100%) | |  | |  |
| Yes | 0 (0%) | 0 (0%) | |  | |  |
| Other related syndromes: |  |  | | 1.000 | |  |
| No | 5382 (99.96%) | 477 (100.00%) | |  | |  |
| Yes | 2 (0.04%) | 0 (0.00%) | |  | |  |
| **Bedside examinations** |  | |  | |  | |
| MMT: |  |  | | <0.001 | |  |
| 1 | 1729 (32.11%) | 53 (11.11%) | |  | |  |
| 2 | 1229 (22.83%) | 94 (19.71%) | |  | |  |
| 3 | 2193 (40.73%) | 199 (41.72%) | |  | |  |
| 4 | 233 (4.33%) | 131 (27.46%) | |  | |  |
| ULBT: |  |  | | <0.001 | |  |
| 1 | 3947 (73.31%) | 217 (45.49%) | |  | |  |
| 2 | 1040 (19.32%) | 129 (27.04%) | |  | |  |
| 3 | 397 (7.37%) | 131 (27.46%) | |  | |  |
| MP: |  |  | | <0.001 | |  |
| 1 | 4732 (87.89%) | 313 (65.62%) | |  | |  |
| 2 | 546 (10.14%) | 117 (24.53%) | |  | |  |
| 3 | 106 (1.97%) | 47 (9.85%) | |  | |  |
| NC | 35.60±3.97 | 36.57±4.18 | | <0.001 | |  |
| LT | 4.44±0.93 | 3.77±1.40 | | <0.001 | |  |
| JD | 3.66±0.55 | 3.59±0.62 | | 0.016 | |  |
| ML | 9.79±1.27 | 9.69±1.37 | | 0.150 | |  |
| CSM: |  |  | | <0.001 | |  |
| 1 | 5264 (97.77%) | 416 (87.21%) | |  | |  |
| 2 | 95 (1.76%) | 43 (9.01%) | |  | |  |
| 3 | 25 (0.46%) | 18 (3.77%) | |  | |  |
| IIG | 4.10±0.83 | 3.19±1.28 | | <0.001 | |  |
| UIL | 0.89±0.19 | 0.89±0.17 | | 0.733 | |  |
| TMD | 9.26±1.42 | 8.67±1.35 | | <0.001 | |  |
| SMD | 16.71±2.25 | 15.59±2.26 | | <0.001 | |  |
| THD | 4.30±1.14 | 3.91±1.03 | | <0.001 | |  |
| HMD | 4.43±0.98 | 4.14±0.96 | | <0.001 | |  |

BMI: body mass index; ASA-PS: American Society of Anesthesiologists Physical Status; MMT: modified Mallampati test; ULBT: upper lip bite test; MP: mandibular protrusion; NC; neck circumference; LT: length of tongue; JD: jaw depth; ML: mandible length; CSM: cervical spine mobility; IIG: inter-incisor gap; UIL: upper incisor length; TMD: thyromental distance; SMD: sternomental distance; THD: thyroid and hyoid distance; HMD: hyomental distance.
