# Supplementary material for: Development and validation of a regression model with nomogram for difficult video laryngoscopy in Chinese population: a prospective, single-center, and nested case-control study
Source: Front Med (Lausanne). 2023 Sep 1;10:1197536. doi: 10.3389/fmed.2023.1197536 (PMC10505806; doi:10.3389/fmed.2023.1197536)
Supplement: Supplementary file 3 [file Table_3.DOCX]

**Supplemental Table S3: Description of the validation set**

|  | **Validation set** | | |
| --- | --- | --- | --- |
|  | **Easy laryngoscopy** | **Difficult laryngoscopy** | **p** |
|  | ***N=2291*** | ***N=222*** |  |
| **Baseline characteristics** |  |  |  |
| Surgical department: |  |  | . |
| ENT | 347 (15.15%) | 23 (10.36%) |  |
| Oral & Maxillofacial Surgery | 1591 (69.45%) | 181 (81.53%) |  |
| Ambulatory ward | 160 (6.98%) | 5 (2.25%) |  |
| Neurosurgery | 30 (1.31%) | 3 (1.35%) |  |
| Ophthalmology | 31 (1.35%) | 1 (0.45%) |  |
| Plastic and Reconstructive Surgery | 126 (5.50%) | 8 (3.60%) |  |
| Others | 6 (0.26%) | 1 (0.45%) |  |
| Chinese nationality: |  |  | 0.321 |
| The Han nationality | 2240 (97.77%) | 220 (99.10%) |  |
| Others | 51 (2.23%) | 2 (0.90%) |  |
| Age | 38.37±15.49 | 47.98±15.76 | <0.001 |
| Gender: |  |  | 0.158 |
| Female | 1254 (54.74%) | 110 (49.55%) |  |
| Male | 1037 (45.26%) | 112 (50.45%) |  |
| Education: |  |  | <0.001 |
| 1 | 532 (23.22%) | 95 (42.79%) |  |
| 2 | 767 (33.48%) | 64 (28.83%) |  |
| 3 | 992 (43.30%) | 63 (28.38%) |  |
| BMI | 22.38±3.57 | 22.72±3.86 | 0.214 |
| Alcohol consumption: |  |  | 0.655 |
| No | 1810 (79.00%) | 172 (77.48%) |  |
| Yes | 481 (21.00%) | 50 (22.52%) |  |
| Smoking: |  |  | 0.142 |
| No | 1825 (79.66%) | 167 (75.23%) |  |
| Yes | 466 (20.34%) | 55 (24.77%) |  |
| Beard: |  |  | . |
| No | 2291 (100.00%) | 222 (100.00%) |  |
| Yes | 0 (0.00%) | 0 (0.00%) |  |
| ASA-PS: |  |  | <0.001 |
| 1 | 1614 (70.45%) | 94 (42.34%) |  |
| 2 | 677 (29.55%) | 128 (57.66%) |  |
| **Medical history** |  |  |  |
| History of cardiovascular diseases: |  |  | <0.001 |
| No | 2008 (87.65%) | 175 (78.83%) |  |
| Yes | 283 (12.35%) | 47 (21.17%) |  |
| History of diabetes: |  |  | 0.143 |
| No | 2224 (97.08%) | 211 (95.05%) |  |
| Yes | 67 (2.92%) | 11 (4.95%) |  |
| History of cranial diseases: |  |  | 0.404 |
| No | 2272 (99.17%) | 222 (100.00%) |  |
| Yes | 19 (0.83%) | 0 (0.00%) |  |
| History of respiratory diseases: |  |  | 0.574 |
| No | 2257 (98.52%) | 218 (98.20%) |  |
| Yes | 34 (1.48%) | 4 (1.80%) |  |
| History of thyroid-related diseases: |  |  | 1.000 |
| No | 2240 (97.77%) | 217 (97.75%) |  |
| Yes | 51 (2.23%) | 5 (2.25%) |  |
| History of liver-related diseases: |  |  | 0.512 |
| No | 2262 (98.73%) | 221 (99.55%) |  |
| Yes | 29 (1.27%) | 1 (0.45%) |  |
| History of gastrointestinal diseases: |  |  | 0.766 |
| No | 2257 (98.52%) | 220 (99.10%) |  |
| Yes | 34 (1.48%) | 2 (0.90%) |  |
| History of spine-related diseases: |  |  | 1.000 |
| No | 2273 (99.21%) | 221 (99.55%) |  |
| Yes | 18 (0.79%) | 1 (0.45%) |  |
| History of urological diseases: |  |  | 0.566 |
| No | 2283 (99.65%) | 221 (99.55%) |  |
| Yes | 8 (0.35%) | 1 (0.45%) |  |
| History of rheumatic and immunological diseases: |  |  | 1.000 |
| No | 2282 (99.61%) | 222 (100.00%) |  |
| Yes | 9 (0.39%) | 0 (0.00%) |  |
| History of gynecological and breast diseases: |  |  | 1.000 |
| No | 2288 (99.87%) | 222 (100.00%) |  |
| Yes | 3 (0.13%) | 0 (0.00%) |  |
| History of allergy: |  |  | 1.000 |
| No | 2288 (99.87%) | 222 (100.00%) |  |
| Yes | 3 (0.13%) | 0 (0.00%) |  |
| History of ENT diseases: |  |  | 0.253 |
| No | 2269 (99.04%) | 222 (100.00%) |  |
| Yes | 22 (0.96%) | 0 (0.00%) |  |
| History of mental illness: |  |  | 1.000 |
| No | 2282 (99.61%) | 222 (100.00%) |  |
| Yes | 9 (0.39%) | 0 (0.00%) |  |
| History of hematologic diseases: |  |  | 0.371 |
| No | 2287 (99.83%) | 221 (99.55%) |  |
| Yes | 4 (0.17%) | 1 (0.45%) |  |
| History of snoring: |  |  | <0.001 |
| No | 1288 (56.22%) | 96 (43.24%) |  |
| Yes | 1003 (43.78%) | 126 (56.76%) |  |
| History of difficult intubation: |  |  | <0.001 |
| No | 2288 (99.87%) | 217 (97.75%) |  |
| Yes | 3 (0.13%) | 5 (2.25%) |  |
| History of radiotherapy: |  |  | <0.001 |
| No | 2242 (97.86%) | 189 (85.14%) |  |
| Yes | 49 (2.14%) | 33 (14.86%) |  |
| History of surgery: |  |  | 0.002 |
| No | 1139 (49.72%) | 86 (38.74%) |  |
| Yes | 1152 (50.28%) | 136 (61.26%) |  |
| History of mandible operation: |  |  | 0.862 |
| No | 2199 (95.98%) | 212 (95.50%) |  |
| Yes | 92 (4.02%) | 10 (4.50%) |  |
| History of rhinitis: |  |  | 0.512 |
| No | 1534 (66.96%) | 154 (69.37%) |  |
| Yes | 757 (33.04%) | 68 (30.63%) |  |
| Nasal congestion: |  |  | 0.003 |
| No | 2081 (90.83%) | 186 (83.78%) |  |
| Left | 52 (2.27%) | 12 (5.41%) |  |
| Right | 99 (4.32%) | 17 (7.66%) |  |
| Bilateral | 59 (2.58%) | 7 (3.15%) |  |
| Head and neck scar: |  |  | 0.004 |
| No | 2257 (98.52%) | 212 (95.50%) |  |
| Yes | 34 (1.48%) | 10 (4.50%) |  |
| History of maxillofacial tumours: |  |  | <0.001 |
| No | 2205 (96.25%) | 185 (83.33%) |  |
| Yes | 86 (3.75%) | 37 (16.67%) |  |
| History of maxillofacial trauma: |  |  | 0.134 |
| No | 2260 (98.65%) | 216 (97.30%) |  |
| Yes | 31 (1.35%) | 6 (2.70%) |  |
| Buck teeth: |  |  | 0.186 |
| No | 2229 (97.29%) | 212 (95.50%) |  |
| Yes | 62 (2.71%) | 10 (4.50%) |  |
| Tongue hypertrophy: |  |  | 0.566 |
| No | 2283 (99.65%) | 221 (99.55%) |  |
| Yes | 8 (0.35%) | 1 (0.45%) |  |
| Laryngeal edema |  |  | 1.000 |
| No | 2291 (100%) | 222 (100%) |  |
| Yes | 0 (0%) | 0 (0%) |  |
| Epiglottis swelling: |  |  | 0.426 |
| No | 2286 (99.78%) | 221 (99.55%) |  |
| Yes | 5 (0.22%) | 1 (0.45%) |  |
| Tonsillar hypertrophy: |  |  | 1.000 |
| No | 2285 (99.74%) | 222 (100.00%) |  |
| Yes | 6 (0.26%) | 0 (0.00%) |  |
| Laryngospasm |  |  | 1.000 |
| No | 2291 (100%) | 222 (100%) |  |
| Yes | 0 (0%) | 0 (0%) |  |
| Bronchospasm |  |  | 1.000 |
| No | 2291 (100%) | 222 (100%) |  |
| Yes | 0 (0%) | 0 (0%) |  |
| Airway obstruction |  |  | 1.000 |
| No | 2291 (100%) | 222 (100%) |  |
| Yes | 0 (0%) | 0 (0%) |  |
| Pneumothorax: |  |  | 1.000 |
| No | 2291 (100.00%) | 222 (100.00%) |  |
| Yes | 0 (0.00%) | 0 (0.00%) |  |
| Thoracic deformity |  |  | 1.000 |
| No | 2291 (100%) | 222 (100%) |  |
| Yes | 0 (0%) | 0 (0%) |  |
| Other related syndromes: |  |  | 1.000 |
| No | 2289 (99.91%) | 222 (100.00%) |  |
| Yes | 2 (0.09%) | 0 (0.00%) |  |
| **Bedside examinations** |  |  |  |
| MMT: |  |  | <0.001 |
| 1 | 703 (30.69%) | 30 (13.51%) |  |
| 2 | 558 (24.36%) | 35 (15.77%) |  |
| 3 | 919 (40.11%) | 84 (37.84%) |  |
| 4 | 111 (4.85%) | 73 (32.88%) |  |
| ULBT: |  |  | <0.001 |
| 1 | 1669 (72.85%) | 102 (45.95%) |  |
| 2 | 445 (19.42%) | 58 (26.13%) |  |
| 3 | 177 (7.73%) | 62 (27.93%) |  |
| MP: |  |  | <0.001 |
| 1 | 2019 (88.13%) | 142 (63.96%) |  |
| 2 | 224 (9.78%) | 55 (24.77%) |  |
| 3 | 48 (2.10%) | 25 (11.26%) |  |
| NC | 35.48±3.94 | 36.21±4.16 | 0.012 |
| LT | 4.42±0.96 | 3.54±1.42 | <0.001 |
| JD | 3.66±0.58 | 3.42±0.57 | <0.001 |
| ML | 9.79±1.16 | 9.54±1.45 | 0.015 |
| CSM: |  |  | <0.001 |
| 1 | 2230 (97.34%) | 196 (88.29%) |  |
| 2 | 51 (2.23%) | 19 (8.56%) |  |
| 3 | 10 (0.44%) | 7 (3.15%) |  |
| IIG | 4.08±0.82 | 3.00±1.30 | <0.001 |
| UIL | 0.89±0.18 | 0.87±0.22 | 0.192 |
| TMD | 9.30±1.41 | 8.51±1.57 | <0.001 |
| SMD | 16.71±2.26 | 15.28±2.21 | <0.001 |
| THD | 4.29±1.13 | 3.91±1.05 | <0.001 |
| HMD | 4.40±0.96 | 4.08±0.97 | <0.001 |

BMI: body mass index; ASA-PS: American Society of Anesthesiologists Physical Status; MMT: modified Mallampati test; ULBT: upper lip bite test; MP: mandibular protrusion; NC; neck circumference; LT: length of tongue; JD: jaw depth; ML: mandible length; CSM: cervical spine mobility; IIG: inter-incisor gap; UIL: upper incisor length; TMD: thyromental distance; SMD: sternomental distance; THD: thyroid and hyoid distance; HMD: hyomental distance.
