# Supplementary material for: Development and validation of a regression model with nomogram for difficult video laryngoscopy in Chinese population: a prospective, single-center, and nested case-control study
Source: Front Med (Lausanne). 2023 Sep 1;10:1197536. doi: 10.3389/fmed.2023.1197536 (PMC10505806; doi:10.3389/fmed.2023.1197536)
Supplement: Supplementary file 4 [file Table_4.DOCX]

**Supplemental Table S4: The comparison between existing data (Caucasian patients) and data from the Chinese Population**

| Study  (Sample size) | Healy 2016  (80801) (1) | Kheterpal 2009  (53041) (2) | Lundstrøm 2009  (103812) (3) | Our study  (8374) |
| --- | --- | --- | --- | --- |
| MMT III-IV (%) | 9597 (11.9) * | 5975 (11.3) * | 6540 (7.1) *  With 11697 missing data | 3943 (47.1) |
| Thick neck (%) | 12834 (15.9) * | 6259 (11.8) * | / | 308 (3.7) |
| Limited TMD (%) | 3548 (4.4) * | 2965 (5.6) * | / | 74 (0.9) |
| Limited MP (%) | 6445 (8.0) * | 4868 (9.2) * | / | 227 (2.7) |
| BMI >35 (%) | / | / | 6003 (5.8) *  With 1705 missing data | 19 (0.2) |
| BMI (Mean ±SD) | / | 28 ±6* | / | 22.4 ±3.6 |
| Beard (%) | / | 5609 (10.6) * | / | 7 (0.1) |

MMT: Modified Mallampati Test; TMD: thyromental distance; MP: Mandibular Protrusion; BMI: body mass index; SD: standard deviation; Thick neck: Neck circumference >43cm (4); *: p <0.001(compared with our study)

1. Healy DW, LaHart EJ, Peoples EE, Jewell ES, Bettendorf RJ, Jr., Ramachandran SK. A Comparison of the Mallampati evaluation in neutral or extended cervical spine positions: a retrospective observational study of >80 000 patients. *Br J Anaesth.* (2016) 116:690-8. doi: 10.1093/bja/aew056.

2. Kheterpal S, Martin L, Shanks AM, Tremper KK. Prediction and outcomes of impossible mask ventilation: a review of 50,000 anesthetics. *Anesthesiology.* (2009) 110:891-7. doi: 10.1097/ALN.0b013e31819b5b87.

3. Lundstrøm LH, Møller AM, Rosenstock C, Astrup G, Gätke MR, Wetterslev J. A documented previous difficult tracheal intubation as a prognostic test for a subsequent difficult tracheal intubation in adults. *Anaesthesia.* (2009) 64:1081-8. doi: 10.1111/j.1365-2044.2009.06057.x.

4. Riad W, Vaez MN, Raveendran R, Tam AD, Quereshy FA, Chung F, et al. Neck circumference as a predictor of difficult intubation and difficult mask ventilation in morbidly obese patients: A prospective observational study. *Eur J Anaesthesiol.* (2016) 33:244-9. doi: 10.1097/eja.0000000000000324.
